# *Nemopilema nomurai* Jellyfish venom treatment leads to alterations in rat cardiomyocytes proteome

**DOI:** 10.1016/j.dib.2015.10.041

**Published:** 2015-11-06

**Authors:** Indu Choudhary, Hyunkyoung Lee, Min-Jung Pyo, Yunwi Heo, Seong Kyeong Bae, Young Chul Kwon, Won Duk Yoon, Changkeun Kang, Euikyung Kim

**Affiliations:** a College of Veterinary Medicine, Gyeongsang National University, Jinju 660-701, Republic of Korea; b Headquarters for Marine Environment, National Fisheries Research & Development Institute, Shiran-ri, Gijang-eup, Gijang-gun, Busan 619-705, Republic of Korea; c Institutes of Agriculture and Life Science, Gyeongsang National University, Jinju, Republic of Korea; d Institute of Animal Medicine, Gyeongsang National University, Jinju 660-701, Republic of Korea

## Abstract

This data article restrains data associated to the Choudhary et al. [1]. *Nemopilema nomurai* Jellyfish venom (NnV) can lead to cardiac toxicity. Here we analyzed the effect of NnV on rat cardiomyocytes cell line H9c2 at the proteome level using two-dimensional gel electrophoresis and matrix-assisted laser desorption/ionization time-of-flight mass spectrometry (MALDI-TOF/MS). This analysis resulted in 34 proteins with differential expression. Here we provide the dataset for the proteins with amplified or reduced level as compare to control.

**Specifications Table**

**Table:** 

Subject area	*Biology*
More specific subject area	*Toxicology*
Type of data	*Table*
How data was acquired	Analysis of the 2-DE gels using Progenesis Same Spots software, protein identification by MALDI-TOF/MS
Data format	*Analyzed*
Experimental factors	To analyze NnV-related proteomic changes, H9c2 cells incubated in the absence or the presence of 3 µg/ml NnV for 6 and 12 h
Experimental features	*After treatment, 2-DE gels were run and then analyzed using* Progenesis Same Spots software and finally identified by MALDI-TOF/MS
Data source location	Specimens of *N. nomurai* jellyfish were collected from Yellow Sea along the coast of Gunsan, South Korea.
Data accessibility	*Data is supplied in this article*

**Value of the data**

• This data includes 34 proteins whose level changed in response to NnV treatment.
• Further studies could be executed from the known proteins related to cardiovascular maintenance or failure.
• In future by targeting these set of proteins one can overcome the lethal cardiovascular effects of NnV.

## 1 Data, experimental design, materials and methods

Data sets comprise of list of 34 proteins which were successfully identified by MALDI-TOF/MS after NnV treatment of H9c2 cell and 2-DE. Among these, 10 proteins were down-regulated and 24 proteins were up-regulated [1]. Differentially expressed (relative spot intensity) proteins and their fold-change along with the up- and down-regulated *p*-values are summarized in Supplementary Table 1 and Supplementary Table 2, respectively.

### 1.1 Sample collection and preparation

Specimens of *N. nomurai* jellyfish were collected from Yellow Sea along the coast of Gunsan, South Korea. Tentacles were dissected and transferred immediately to the laboratory in ice for further preparation. Nematocysts were isolated as described previously [2]. Briefly to remove any debris, dissected tentacles were rinsed with cold sea water, and then 3 volumes (v/v) of cold sea water was added and kept on shaker for 1 day at 4 °C. After that tentacle free sea water was centrifuged at 1000*g* for 5 min, pellet was collected and washed thrice with sea water. Sedimented tentacles were further autolysed in fresh sea water at 4 °C for 1 day as described above and autolysis process was repeated for 3–4 days. Finally the settled nematocysts were collected and washed several times with sea water. Nematocysts were harvested at 500*g* for 5 min and pellets (nematocysts) were lyophilized and stored at −20 °C until further use.

### 1.2 Venom extraction and preparation

Venom was extracted from the freeze-dried nematocysts using the technique described by Carrette and Seymour [3] with slight modification. In brief, venom was extracted from 50 mg of lyophilized nematocyst using glass beads (approximately 8000 beads; 0.5 mm in diameter) and 1 ml of phosphate buffered saline (PBS, pH 7.4, kept at 4 C°). These samples were shaken in a mini bead mill at 3000 rpm for 30 s, repeated 10 times with intermittent cooling on ice. The venom extracts were then transferred to a new eppendorf tube and centrifuged (22,000*g*) at 4 °C for 30 min. This supernatant was used as NnV for the present study. Protein concentration of the venom was determined by Bradford assay (Bio-Rad, C.A, USA) [4] and the venom was used based on its protein concentration.

### 1.3 Cell culture

The rat cardiomyocytes cell line H9c2 was purchased from the American Type Culture Collection (ATCC) (Manssas, 7VA) and grown in Dulbecco׳s modified Eagle׳s medium (DMEM) supplemented with 10% heat inactivated fetal bovine serum (FBS), 100 µg/ml penicillin–streptomycin–amphotericin B solution. The cells were maintained in a humidified incubator at 37 °C, with 5% CO_2_ and their medium was replaced every 2–3 days.

### 1.4 Protein extraction and sample preparation

For protein extraction, the H9c2 cells at 70% confluence were used, the cell lines were subsequently treated with 3 µg/ml concentration of NnV for 6 h and 12 h. After the incubation the cells were washed with ice cold 1× phosphate buffered saline (PBS) and scrapped in 2-DE lysis buffer (7 M Urea, 2 M thiourea, 4% (w/v) CHAPS) (3-[(3-cholamidopropyl) dimethylammonium]-1-propane sulfonate), 10% DTT, 0.5% IPG (immobilized pH gradient) buffer and 1% proteinase inhibitor. Samples were vortexed for 30 min at 4 °C and insoluble parts were removed by centrifugation (14,000*g*, 15 min, 4 °C). To cell lysis supernatant, equal volume of 20% TCA (trichloroacetic acid) was added, the mixture was incubated for 30 min on ice and centrifuged at 14,000*g* for 15 min at 4 °C. The supernatant was removed and pellets were washed twice with 200 µl of ice cold acetone and centrifuged as above. Then the pellets were vacuum dried for 10–15 min. Subsequently, dried pellets were dissolved in a sample buffer containing (7 M urea, 2 M thiourea, 4% (w/v) CHAPS). Protein concentration of the samples was determined by using Bradford assay.

### 1.5 Two-dimensional gel electrophoresis and image analysis

To proceed 2-DE, 225 µg of protein sample was diluted to 340 µl with a rehydration buffer containing (7 M urea, 2 M thiourea, 4% (w/v) CHAPS, 10 mg/ml DTT, 1% pharmalytes (pH 4–7) and few grains of bromophenol blue). Further protein samples were applied to Immobiline^TM^ Drystrip (pH 4–7) 18 cm for rehydration process, which was carried out overnight at room temperature. Isoelectric focusing was performed using Ettan IPGphor system (GE Healthcare) and the protocol includes 8 steps: 50 V for 1:00 h, 200 V for 1:00 h, 500 V for 0:30 h, gradient 4000 V for 0:30 h, 4000 V for 1:00 h, gradient 10,000 V for 1:00 h, 10,000 V for 13:00 h and 50 V 3:00 h at 20 °C. During two step equilibration, firstly strips were equilibrated in a mixture of 50 mM Tris–HCl (pH 8.8), 6 M urea, 30% glycerol, 2% SDS and 0.01% bromophenol blue containing 1% w/v DTT and then DTT was replaced with 2.5% w/v iodoacetamide in second step. For second dimension, equilibrated strips were subsequently positioned on the top of 12% SDS polyacrylamide gels (18 cm×20 cm×1.5 mm). The strips were sealed with 0.5% (w/v) agarose prepared in a SDS running buffer containing trace amount of bromophenol blue. The SDS gels were run at 20 mA/gel at 20 °C until the dye front reached the bottom of the gels.

All the gels were run in quadruplicate, protein spots were visualized by silver staining. The stained gels were scanned by Epson perfection V 700 photo scanner (Epson) and image analysis was performed using Progenesis Same Spots software (Nonlinear Dynamics, Newcastle UK). Automated image analysis was retrieved by manual editing (which includes, spot detection, background subtraction, spot matching and total spot volume normalization) to co-detect differentially expressed protein spots. NnV induced differentially expressed proteins with statistical significance level of *p*≤0.05 based on one way ANOVA (analysis of variance) analysis were selected for MALDI-TOF/MS analysis.

The differentially expressed proteins were manually excised from 2-DE gels. In-gel digestion of the proteins was performed as follows, the gel pieces were washed with destain solution containing 30 mM potassium ferricyanide and 100 mM Na_2_S_2_O_3_ (1:1, v/v) for 10 min. Subsequently, the gel pieces were dehydrated in 100% ACN for 10 min, and dried in lypholizer equipment. Then reduction was carried out by adding reduction solution (10 mM DTT in 100 mM ammonium bicarbonate) for 45 min at 56 °C. After incubation reduction solution was removed and the alkylation solution (100 mM iodoacetamide in 100 mM ammonium bicarbonate) was added to the samples and incubated for 45 min at room temperature. After that the gel pieces were incubated in trypsin (Promega) at a final concentration of 20 ng/µl in 5–10 µl of 50 mM NH_4_HCO_3_ on ice for 45 min. After removing trypsin, adequate amount of 50 mM NH_4_HCO_3_ was added to cover the gel pieces and then tubes with gel pieces were incubated overnight at 37 °C. The tryptic peptide mixture was extracted with extraction buffer containing 100% ACN and 50% TFA and was concentrated in a vacuum centrifuge.

### 1.6 MALDI-TOF/MS analysis and database searching

The peptide extracts were re-dissolved in 1 µl of HCCAs matrix solution (α-acyano-4-hydroxycinnamic acid) together with 1 µl of extraction buffer and targeted onto a freshly cleaned MALDI-TOF plate. The mass spectrometry (MS) spectra were recorded by using a Voyager-DE STR mass spectrometer (Applied Biosystems, Franklin Lakes, NJ, USA). The spectra were acquired by reflection/delayed extraction mode. The spectra were recorded over a mass range of 800–3000 Da. Swiss prot and NCBI (National Center for Biotechnology Information) database were searched using online Mascot server (Matrix science http://www.matrixscience.com). Peptide mass finger printing was performed using following settings: taxonomy as *Rattus norvegicus* (rattus), peptide mass tolerance of ±100 ppm for the fragment ions, trypsin with one cleavage is allowed, fixed modification with alkylation of cysteine by carbamidomethyl, oxidation of methionine as a variable modification during the search of the peptides.

## Appendix A Supplementary material

**Supplementary Table 1.** The proteins with increment in relative abundance after NnV treatment. Proteins with ≥1.5 fold change and with statistically significance *p*-value≤ 0.05 were considered. Fold change and *p*-value of individual candidate protein is mentioned.

**Supplementary Table 2.** The proteins with declination in relative abundance after NnV treatment. Protein fold change and *p*-value of individual candidate protein is mentioned.
