# Supplementary material for: Nemopilema nomurai Jellyfish venom treatment leads to alterations in rat cardiomyocytes proteome
Source: Data Brief. 2015 Nov 6;5:884–7. doi: 10.1016/j.dib.2015.10.041 (PMC4669470; doi:10.1016/j.dib.2015.10.041)
Supplement: Supplementary file 1 — Supplementary Table 1. The proteins with increment in relative abundance after NnV treatment. Proteins with ≥1.5 fold change and with statistically significance p-value≤ 0.05 were considered. Fold change and p-value of individual candidate protein is mentioned. [file mmc1.ppt]

## Slide 1
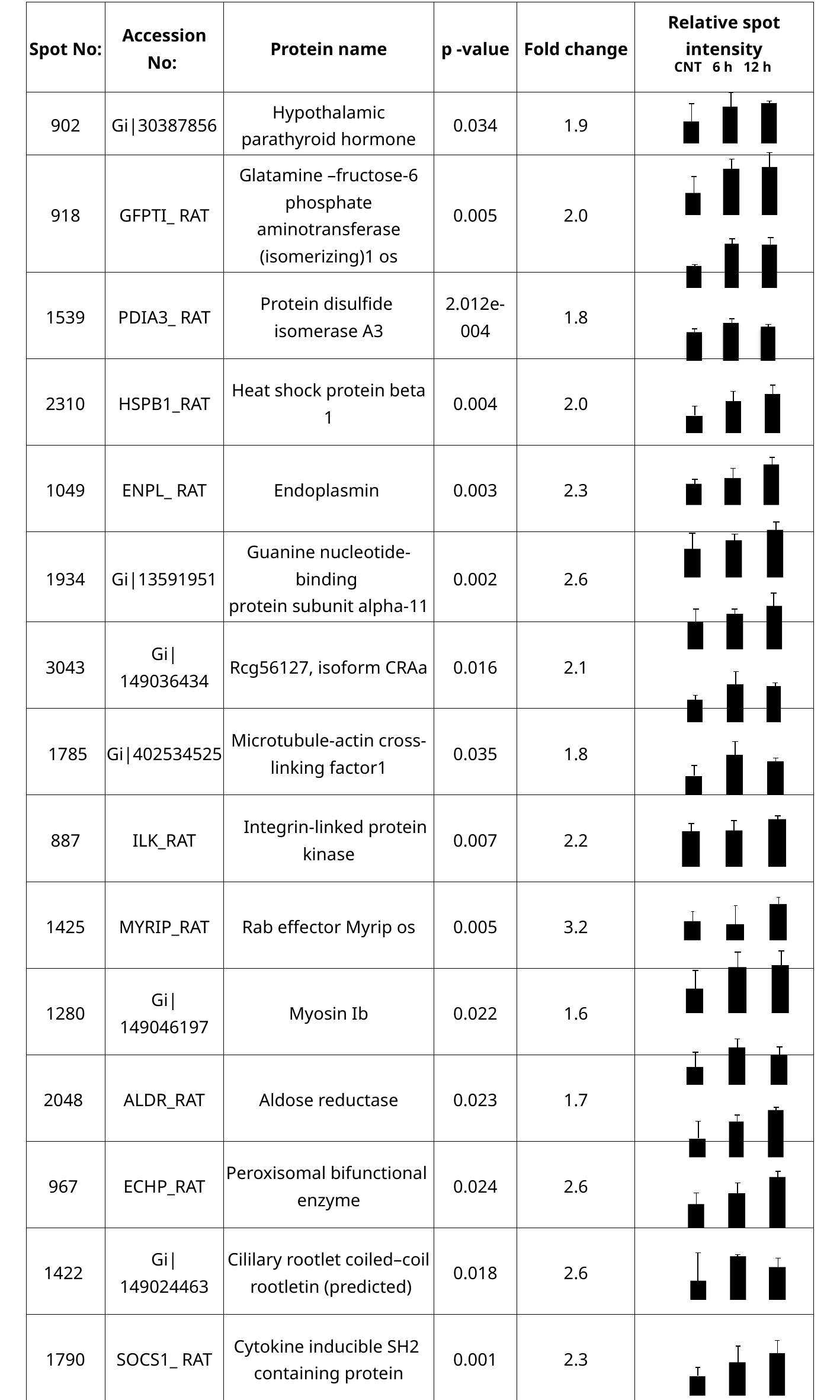

| Spot No: | Accession No: | Protein name | p -value | Fold change | Relative spot intensity |
| --- | --- | --- | --- | --- | --- |
| 902 | Gi|30387856 | Hypothalamic parathyroid hormone | 0.034 | 1.9 | |
| 918 | GFPTI\_ RAT | Glatamine –fructose-6 phosphate aminotransferase (isomerizing)1 os | 0.005 | 2.0 | |
| 1539 | PDIA3\_ RAT | Protein disulfide isomerase A3 | 2.012e-004 | 1.8 | |
| 2310 | HSPB1\_RAT | Heat shock protein beta 1 | 0.004 | 2.0 | |
| 1049 | ENPL\_ RAT | Endoplasmin | 0.003 | 2.3 | |
| 1934 | Gi|13591951 | Guanine nucleotide- binding protein subunit alpha-11 | 0.002 | 2.6 | |
| 3043 | Gi| 149036434 | Rcg56127, isoform CRAa | 0.016 | 2.1 | |
| 1785 | Gi|402534525 | Microtubule-actin cross- linking factor1 | 0.035 | 1.8 | |
| 887 | ILK\_RAT | Integrin-linked protein kinase | 0.007 | 2.2 | |
| 1425 | MYRIP\_RAT | Rab effector Myrip os | 0.005 | 3.2 | |
| 1280 | Gi| 149046197 | Myosin Ib | 0.022 | 1.6 | |
| 2048 | ALDR\_RAT | Aldose reductase | 0.023 | 1.7 | |
| 967 | ECHP\_RAT | Peroxisomal bifunctional enzyme | 0.024 | 2.6 | |
| 1422 | Gi| 149024463 | Cililary rootlet coiled–coil rootletin (predicted) | 0.018 | 2.6 | |
| 1790 | SOCS1\_ RAT | Cytokine inducible SH2 containing protein | 0.001 | 2.3 | |
| 1792 | PALM\_RAT | Paralemmin -1 | 9.306e-004 | 2.4 | |
| 1433 | MYH7\_RAT | Myosin 7 | 0.040 | 2.0 | |
| 1286 | ANXA2\_RAT | ANNEXIN A2 | 0.016 | 2.0 | |
CNT 6 h 12 h

## Slide 2
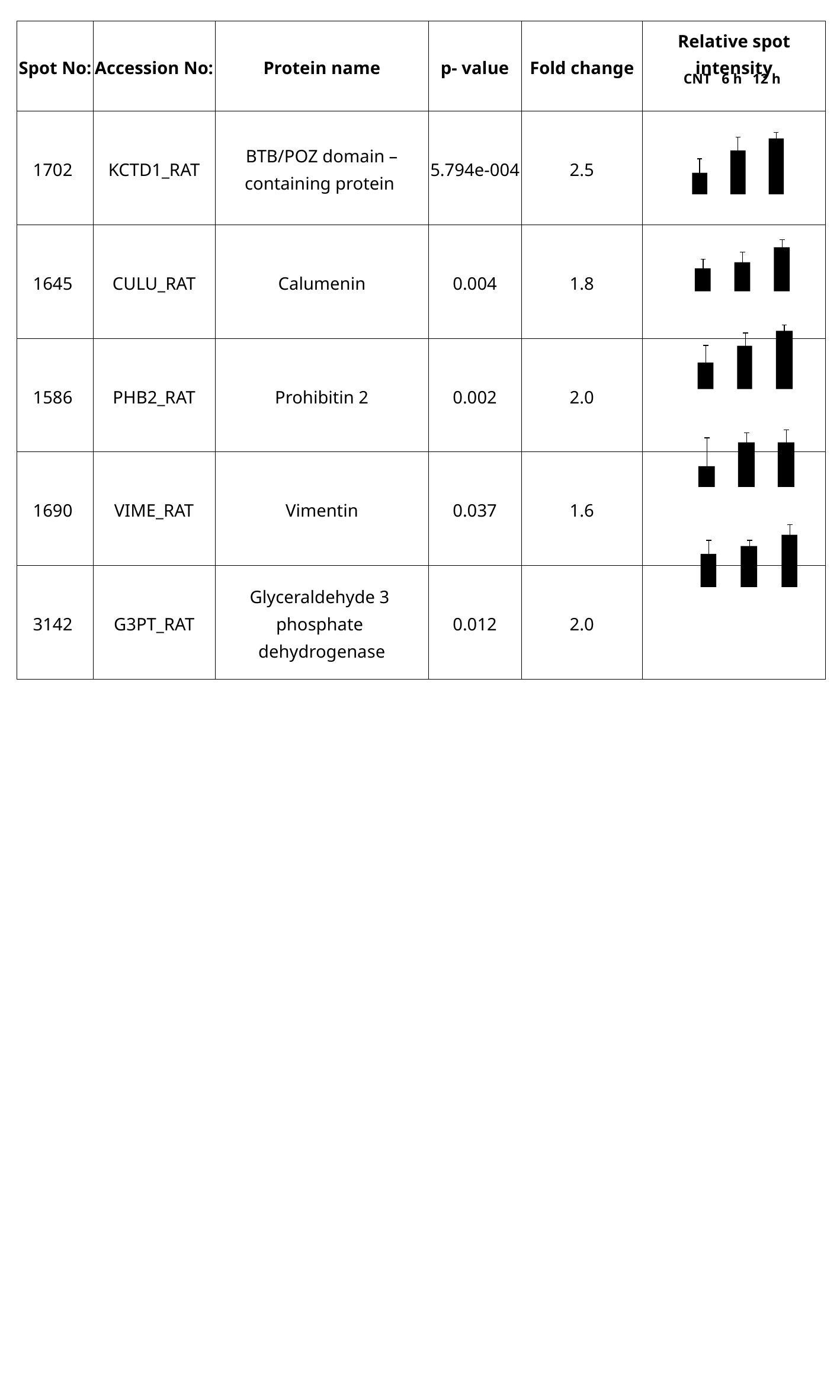

| Spot No: | Accession No: | Protein name | p- value | Fold change | Relative spot intensity |
| --- | --- | --- | --- | --- | --- |
| 1702 | KCTD1\_RAT | BTB/POZ domain – containing protein | 5.794e-004 | 2.5 | |
| 1645 | CULU\_RAT | Calumenin | 0.004 | 1.8 | |
| 1586 | PHB2\_RAT | Prohibitin 2 | 0.002 | 2.0 | |
| 1690 | VIME\_RAT | Vimentin | 0.037 | 1.6 | |
| 3142 | G3PT\_RAT | Glyceraldehyde 3 phosphate dehydrogenase | 0.012 | 2.0 | |
CNT 6 h 12 h
