# Supplementary material for: Nemopilema nomurai Jellyfish venom treatment leads to alterations in rat cardiomyocytes proteome
Source: Data Brief. 2015 Nov 6;5:884–7. doi: 10.1016/j.dib.2015.10.041 (PMC4669470; doi:10.1016/j.dib.2015.10.041)
Supplement: Supplementary file 2 — Supplementary Table 2. The proteins with declination in relative abundance after NnV treatment. Protein fold change and p-value of individual candidate protein is mentioned. [file mmc2.ppt]

## Slide 1
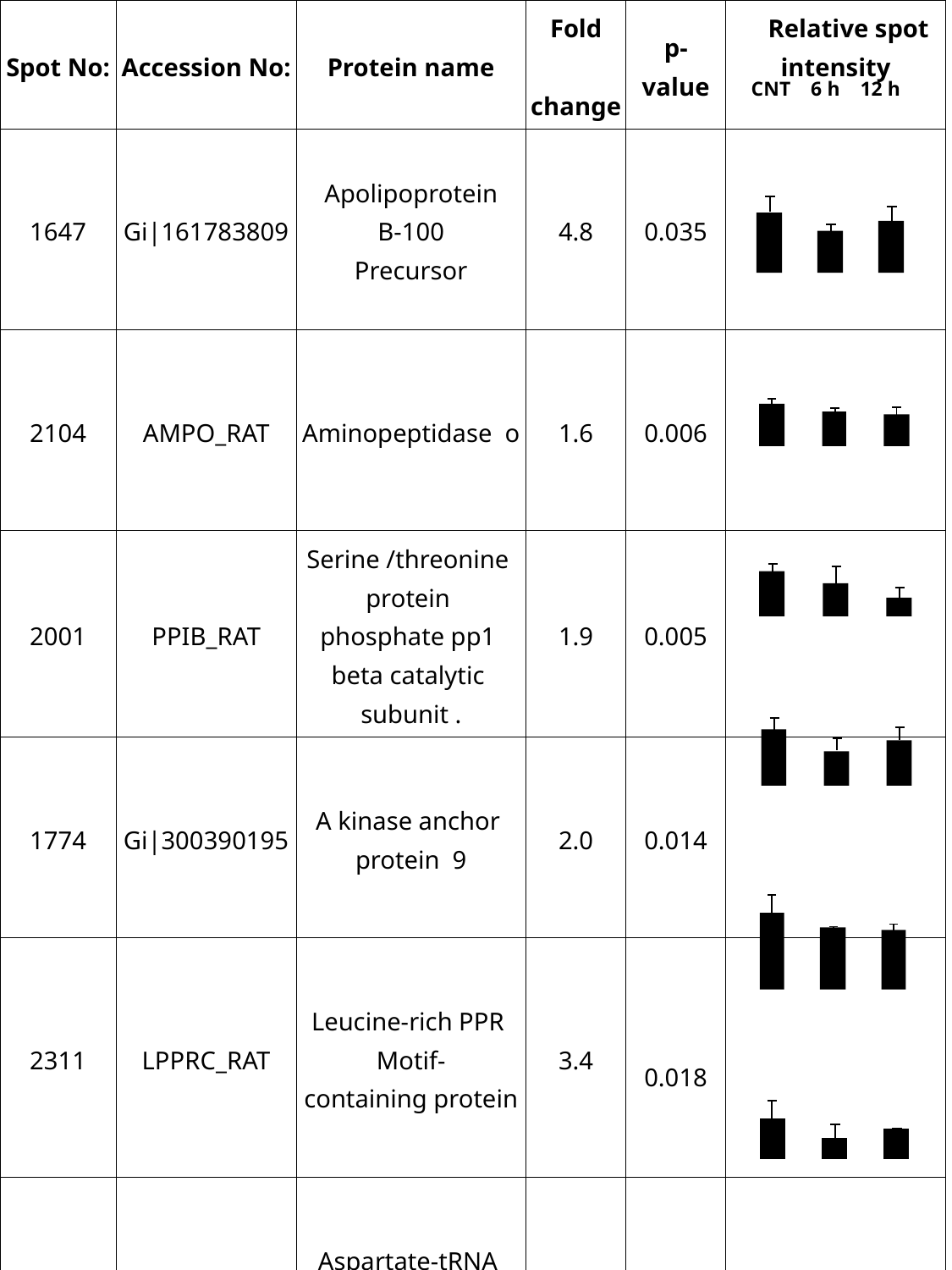

| Spot No: | Accession No: | Protein name | Fold change | p- value | Relative spot intensity |
| --- | --- | --- | --- | --- | --- |
| 1647 | Gi|161783809 | Apolipoprotein B-100 Precursor | 4.8 | 0.035 | |
| 2104 | AMPO\_RAT | Aminopeptidase o | 1.6 | 0.006 | |
| 2001 | PPIB\_RAT | Serine /threonine protein phosphate pp1 beta catalytic subunit . | 1.9 | 0.005 | |
| 1774 | Gi|300390195 | A kinase anchor protein 9 | 2.0 | 0.014 | |
| 2311 | LPPRC\_RAT | Leucine-rich PPR Motif- containing protein | 3.4 | 0.018 | |
| 1618 | SYDC\_RAT | Aspartate-tRNA ligase | 2.3 | 0.036 | |
CNT 6 h 12 h

## Slide 2
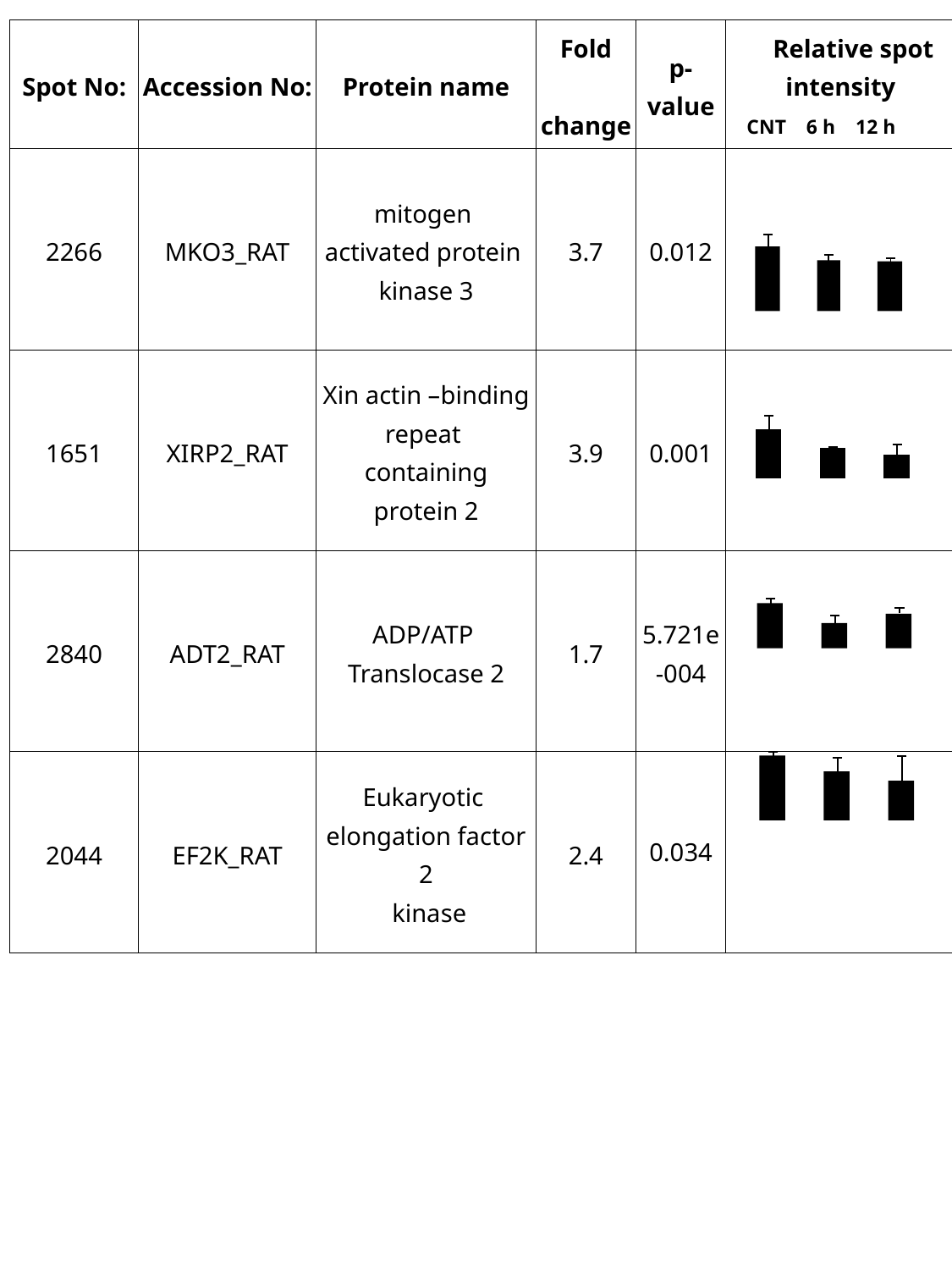

| Spot No: | Accession No: | Protein name | Fold change | p- value | Relative spot intensity |
| --- | --- | --- | --- | --- | --- |
| 2266 | MKO3\_RAT | mitogen activated protein kinase 3 | 3.7 | 0.012 | |
| 1651 | XIRP2\_RAT | Xin actin –binding repeat containing protein 2 | 3.9 | 0.001 | |
| 2840 | ADT2\_RAT | ADP/ATP Translocase 2 | 1.7 | 5.721e-004 | |
| 2044 | EF2K\_RAT | Eukaryotic elongation factor 2 kinase | 2.4 | 0.034 | |
CNT 6 h 12 h
